# Dapagliflozin Alleviates Hepatic Steatosis by Restoring Autophagy via the AMPK-mTOR Pathway

**DOI:** 10.3389/fphar.2021.589273

**Published:** 2021-05-17

**Authors:** Liuran Li, Qinghua Li, Wenbin Huang, Yibing Han, Huiting Tan, Min An, Qianru Xiang, Rui Zhou, Li Yang, Yanzhen Cheng

**Affiliations:** ^1^ Department of Endocrinology, Zhujiang Hospital, Southern Medical University, Guangzhou, China; ^2^ Department of Hepatobiliary Surgery II, Zhujiang Hospital, Southern Medical University, Guangzhou, China; ^3^ Department of Pathology, School of Basic Medical Sciences, Southern Medical University, Guangzhou, China; ^4^ Department of Nutrition, Zhujiang Hospital, Southern Medical University, Guangzhou, China

**Keywords:** NAFLD, AMPK, mTOR, autophagy, dapagliflozin

## Abstract

As a newly approved oral hypoglycaemic agent, the sodium-glucose cotransporter 2 (SGLT2) inhibitor dapagliflozin, which is derived from the natural product phlorizin can effectively reduce blood glucose. Recent clinical studies have found that dapagliflozin alleviates non-alcoholic fatty liver disease (NAFLD), but the specific mechanism remains to be explored. This study aimed to investigate the underlying mechanism of dapagliflozin in alleviating hepatocyte steatosis *in vitro* and *in vivo*. We fed the spontaneous type 2 diabetes mellitus rats with high-fat diets and cultured human normal liver LO2 cells and human hepatocellular carcinoma HepG2 cells with palmitic acid (PA) to induce hepatocellular steatosis. Dapagliflozin attenuated hepatic lipid accumulation both *in vitro* and *in vivo*. In Zucker diabetic fatty (ZDF) rats, dapagliflozin reduced hepatic lipid accumulation via promoting phosphorylation of acetyl-CoA carboxylase 1 (ACC1), and upregulating lipid *β*-oxidation enzyme acyl-CoA oxidase 1 (ACOX1). Furthermore, dapagliflozin increased the expression of the autophagy-related markers LC3B and Beclin1, in parallel with a drop in p62 level. Similar effects were observed in PA-stimulated LO2 cells and HepG2 cells. Dapagliflozin treatment could also significantly activated AMPK and reduced the phosphorylation of mTOR in ZDF rats and PA-stimulated LO2 cells and HepG2 cells. We demonstrated that dapagliflozin ameliorates hepatic steatosis by decreasing lipogenic enzyme, while inducing fatty acid oxidation enzyme and autophagy, which could be associated with AMPK activation. Moreover, our results indicate that dapagliflozin induces autophagy via the AMPK-mTOR pathway. These findings reveal a novel clinical application and functional mechanism of dapagliflozin in the treatment of NAFLD.

## Introduction

Non-alcoholic fatty liver disease (NAFLD), the most common liver disease worldwide, is characterized by the hepatic fat accumulation in patients without alcohol abuse (Angulo, 2002). Metabolic syndromes, such as obesity and hyperglycaemia, predispose patients to developing severe NAFLDs, including nonalcoholic steatohepatitis (NASH), liver cirrhosis, and hepatocellular carcinoma (Lu et al., 2018). In patients with type 2 diabetes mellitus (T2DM), the prevalence rate of NAFLD is approximately 70%, among which 20% develops advanced fibrosis, revealing the high correlation between diabetes and NAFLD (Bril et al., 2016). In fact, NAFLD is only one outcome of multisystem diseases, in which the most frequent causes of high morbidity and mortality are cardiovascular diseases, extrahepatic malignant tumors and liver-related complications (Adams et al., 2017). At present, the common measures for NAFLD treatment are restricted to diet adjustment, exercise and bariatric surgery, due to lacking of effective and safe medications. Therefore, a pharmacological treatment for NAFLD is urgently required.

Dapagliflozin, a sodium-glucose cotransporter 2 (SGLT2) inhibitor, is a novel antidiabetic agent that is approved for the treatment of T2DM by based on its ability to inhibit SGLT2-mediated renal glucose reabsorption. In addition to providing effective glycaemic control, dapagliflozin help decrease body weight, reduce rate of cardiovascular death, and possibly slow the progression of diabetic kidney diseases (Dhillon, 2019). Dapagliflozin has been reported to alleviate hepatic steatosis in patients with type 2 diabetes and NAFLD; however, whether the improvement in liver steatosis is related to the reduction in body weight by dapagliflozin cannot be ruled out (Kurinami et al., 2018; Shimizu et al., 2019). A recent study suggested that dapagliflozin attenuated SGLT2 expression, which could prevent excessive glucose absorption and increase AMPK phosphorylation in HFD-induced obese mice or OA-stimulated HuS-E/2 cells (Chiang et al., 2020), but the explicit mechanism has not yet been fully clarified. Thus, whether dapagliflozin can moderate hepatic steatosis independent of hypoglycaemic control and body weight loss requires further investigation.

Acetyl-CoA carboxylase 1 (ACC1), a rate-controlling enzyme in *de novo* lipogenesis, plays a crucial role in fatty acid metabolism. Inhibition of ACC1 can significantly reduce hepatic steatosis and hepatic insulin resistance (Brownsey et al., 1997; Goedeke et al., 2018). Acyl-CoA oxidase 1 (ACOX1), a rate-limiting enzyme in the peroxisomal β-oxidation pathway, promotes catabolism of very long–chain fatty acids. ACOX1-deficient mice exhibit spontaneous hepatic steatosis and steatohepatitis (Sheridan et al., 2011; Moreno-Fernandez et al., 2018). Hepatic lipid accumulation is caused by an imbalance between lipid synthesis and lipid degradation, which are mediated through several pathways, such as increased *de novo* lipogenesis and reduced fatty acid oxidation. AMPK is a vital energy sensor that regulates hepatic lipid metabolism (Madiraju et al., 2016). mTOR, a crucial downstream target of AMPK, negatively regulates autophagy (Kim and Guan., 2015). It was reported that the hepatic autophagy was suppressed by inhibiting AMPK, which caused the development of hepatic steatosis (You et al., 2004). Although the pathogenesis of NAFLD is elusive, increasing evidence suggests that impaired autophagy plays a vital role in the development of NAFLD. In the present study, we investigated the protective effect of the SGLT2 inhibitor dapagliflozin in alleviating NAFLD both *in vivo* and *in vitro*, and further clarified the role of AMPK activation in ameliorating hepatic lipid accumulation and the involvement of the AMPK-mTOR signaling pathway in autophagy induction.

## Materials and Methods

### Antibodies and Reagents

Protein expression was assessed by immunoblot analysis of cell lysates (20–60 μg) in RIPA buffer in the presence of the following antibodies: including anti-p-AMPK (Affinity, AF3423), anti-AMPK (Affinity, AF6423), anti-p-ACC1 (Cell Signaling Technology, 11818s), anti-ACC1(Cell Signaling Technology, 4190s), anti-ACOX1 (Santa Cruz Biotechnology, sc-517306), anti-p-mTOR (Affinity, AF3308), anti-mTOR (Affinity, AF6308), anti-SGLT2 (Abcam, ab37296), anti-LC3B (Cell Signaling Technology, 12741S), anti-Beclin1 (Cell Signaling Technology, 4122s), anti-p62/SQSTM1 (Proteintech, 18420-1-AP), and anti-GAPDH (Abcam, ab8245).

Unless otherwise specified, all chemicals were purchased from Sigma-Aldrich (St. Louis, MO, United States). Dulbecco's modified Eagle’s medium (DMEM), fetal bovine serum (FBS, Gibco) were obtained from Gibco; palmitate (PA) was obtained from Sigma-Aldrich (P9767); compound C (Comp C) was purchased from AbMole (M2238), chloroquine (CQ, S4157) and dapagliflozin (S1548) were bought from Selleck; and Oil Red O and triglyceride detection kit (G1262; BC0625) were obtained from Solarbio.

### Animal Model

Eight-week-old male Zucker Diabetic Fatty (ZDF, fa/fa) rats and Zucker lean (ZL, fa/+) rats were purchased from the Laboratory Animal Center of Vital River [Beijing, China; license number, SYXK (Yue) 2011–0,074]. Five ZL rats and 10 male ZDF rats, at 8 weeks of age, were fed with a high-fat diet Purina #5008 for 4 weeks to induce diabetes. At the age of 12 weeks, the ZDF rats were randomly separated into two groups (*n* = 5 in each group) including: the diabetic control group (ZDF) and the diabetic group treated with dapagliflozin (ZDF + Dapa). In addition, the rats in ZL group were used as the non-diabetic controls. High-fat diet Purina #5008 was provided from 8 weeks of age to the end of the experiment. The rats in the ZDF + Dapa group were administered with dapagliflozin at a dosage of 1.0 mg/kg/day using intragastric gavage, while the ZL group and the ZDF group were treated with a vehicle via gavage as control. The interventions had been maintained for 9 weeks. Glucose levels of all the rats in different groups were measured every week. At the age of 21 weeks, all rats were anesthetized and sacrificed, blood was collected via the tail vein, and liver tissues were weighed at once after sampling and then stored at −80°C for subsequent measurements. All animal experimental procedures were performed with the approval of the Southern Medical University Animal Care and Use Committee in accordance with established ethical guidelines for animal studies (Resolution No. L2015039).

### Oral Glucose Tolerance Tests

Oral glucose tolerance tests (2 g/kg of body weight) were performed on overnight fasted rats using a glucometer, and the blood samples were obtained from the tail vein at 0, 30, 60, and 120 min to measure blood glucose levels. Ultimately, the area under the curve (AUC) of blood glucose was calculated by the trapezoidal method (AUC = 1/4*fasting glucose +1/2*30-min glucose +3/4*60-min glucose +1/2*120-min glucose).

### Cell Viability Assay

Cell viability assays of HepG2 cells and LO2 cells were carried out using Cell Counting Kit 8 (CCK8) (Dojindo; Kumamoto, Japan). Cells were plated into 96-well plates at a density of 1 × 10^4 cells per well and cultured overnight. Then, the culture medium was replaced by DMEM supplemented with PA (0.3 mM) and dapagliflozin (0, 10, 20, 40, 80, and 100 μg/ml). After incubation for 24 h, 10 μL CCK-8 reagent and 90 μL DMEM were added to each well for an additional 2 h of cultivation and the absorbance value was analyzed with a microplate reader at the wavelength of 450 nm.

### Cell Culture and Treatments

Immortalized normal human hepatocyte-derived liver cells (LO2) and human hepatocellular carcinoma cells (HepG2) were obtained from Shanghai Institute of Cellular Biology of Chinese Academy of Sciences. When cultured in DMEM supplemented with 10% FBS at 37°C in a humidified incubator containing 5% CO2, the cells were treated with 0.3 mM PA for 24 h to create the hepatocyte steatosis model *in vitro*. Then, hepatocytes were exposed to dapagliflozin at a concentration of 20 μM with or without chloroquine for 24 h. To study the effect of AMPK inhibition on autophagy, cells were treated with 10 μM compound C (Comp C) for 24 h.

### Western Blot Analysis

HepG2 cells, LO2 cells and rat liver tissues were extracted in RIPA lysis buffer with protease inhibitor (Beyotime, Biotechnology). Phosphatase inhibitor was added to detect phosphorylated proteins. The protein concentrations of the lysates were quantified using the BCA Protein Assay Kit (Beyotime). Equal amounts of proteins were size-separated on a 10% SDS-polyacrylamide gel and then electroblotted onto PVDF membranes. Membranes were blocked by using a blocking reagent (0.1% Tween 20 and 5% Bovine Serum Albumin in TBS) for 1 h and subsequently incubated with specific primary antibodies (1:1,000) against LC3B, AMPK, p-AMPK, p62, Beclin1, ACC1, p-ACC1, ACOX1, p-mTOR, mTOR, SGLT2, and GAPDH overnight at 4 °C. After incubation with the secondary antibodies (1:10,000) for 1 h at room temperature, the signals were developed with an ECL luminescent kit and detected using enhanced chemiluminescence detection system (Pierce, Rockford, IL). The protein bands were analyzed densitometrically by ImageJ, and GAPDH protein was used as an internal control.

### RNA Isolation and Quantitative Real-Time PCR Analysis

Total RNA was extracted from liver tissues and cells using TRIzol reagent and reverse-transcribed into cDNA according to the manufacturer’s protocols. A quantitative PCR (qPCR) analysis was performed using SYBR Green PCR master mix (Applied Biosystems; Foster City, CA) on a 7,500 Fast Real-Time PCR system to examine the expression of related genes. The sequences of the primers used in this study are listed in (Supplementary Table S1). The expression levels of the target genes were normalized to that of GAPDH, and the ΔΔcycle threshold method was used for the quantitative analysis. All reagents in this study including those for RNA preparation, reverse transcription and quantitative real-time PCR analysis were purchased from Takara (Japan).

### Immunofluorescence Staining

Immunofluorescence staining was performed according to standard protocols. Cells were fixed with 4% paraformaldehyde for 10 min, permeabilized in 0.25% Triton X-100 for 15 min and blocked with 5% normal goat serum for 1 h. Samples were incubated with primary antibodies against SGLT2 (1:100) and LC3B (1:100) at 4°C overnight followed by the incubation with secondary antibodies (1:100; Jackson Laboratories) for 1 h at room temperature, and the staining of 4′,6-diamidino-2-phenylindole (DAPI; 1:100; Invitrogen) for 4min. All cells were observed and captured under an Olympus FluoView FV1000 confocal microscope (Olympus, Hamburg, Germany). For the quantitative analysis, the average score of selected areas was calculated using Image Pro Plus 6.0 software (Media Cybernetics, Inc., Rockville, MD, United States).

### Intracellular Lipid Droplets Staining

The accumulation of intracellular lipid droplets was detected by BODIPY 493/503 staining. Hepatocytes were treated with the indicated concentrations of PA and dapagliflozin for 24 h. Subsequently, the cells were fixed with 4% paraformaldehyde and stained with 1 μg/ml BODIPY 493/503 for 30 min at 37°C. Cell images and fluorescence intensity were visualized and quantified using an Olympus FluoView FV1000 confocal microscope.

### Oil Red O Staining and Cellular Triglyceride Assays

Lipid droplets were visualized and quantified by Oil Red O (ORO) staining. According to the instructions, the cells were fixed with ORO fixative for 20min, and stained with newly prepared ORO staining solution for 20 min. After washing with 60% isopropanol, they were restained by Mayer hematoxylin for 2 min. Then, red-stained lipid droplets were subsequently observed and imaged using a light microscope (Olympus, Japan) at 400 × magnification. To quantify the intracellular lipid accumulation, the TG content in HepG2 cells and LO2 cells was measured using a Triglyceride Content Detection Kit according to the manufacturer's recommended protocols.

### Liver Histopathological Examination

For Oil Red O staining, 8-μm-thick frozen sections were prepared and stained with freshly diluted Oil Red O staining solution for 20 min. After washed with 60% isopropanol, the sections were re-stained by Mayer hematoxylin for 2 min. The histological features of the samples were observed and imaged using a light microscopy. For hematoxylin and eosin (H&E) staining, the liver tissue was fixed in 4% formalin for 24 h, and then maintained in 70% alcohol for subsequent processing in paraffin for the histological studies. Paraffin sections were cut at 3 μm before paraffin removal. Then slices were obtained and stained with hematoxylin-eosin reagents.

### Immunohistochemistry Staining (IHC)

IHC was used to detect the expression of proteins in 3 μm sections from formalin-fixed and paraffin-embedded tissue specimens as previously described (Tang et al., 2019)*.* The tissues were incubated with primary antibodies (1:200) against LC3B, Beclin1, p62, and SGLT-2 at 4°C overnight, followed by incubation with the secondary antibody. Then the sections were stained with a 3,3′-diaminobenzidine solution for 3 min and counterstained with Mayer’s hematoxylin. Photomicrographs were captured under an optical microscope at 400 × magnification. Image-Pro Plus 6.0 software was used to select the same brown-yellow color as the unified standard for judging the positiveness of all photos, and analyze each photo to get the cumulative optical density value (IOD) of each photo and the pixel area of to be tested (AREA). The area density value (IOD/AREA) was obtained, and a larger value corresponded to a higher positive expression level.

### Statistical Analysis

The results are expressed as the mean ± SEM. Comparisons between two groups were assessed using a *t*-test. Statistical analyses were performed using SPSS 20.0 statistical software and GraphPad Prism 6.02. Statistical significance was defined as a *p* value of <0.05.

## Results

### Dapagliflozin Improves Glucose Intolerance and Regulates Lipid Metabolism in ZDF Rats

**FIGURE 1:**
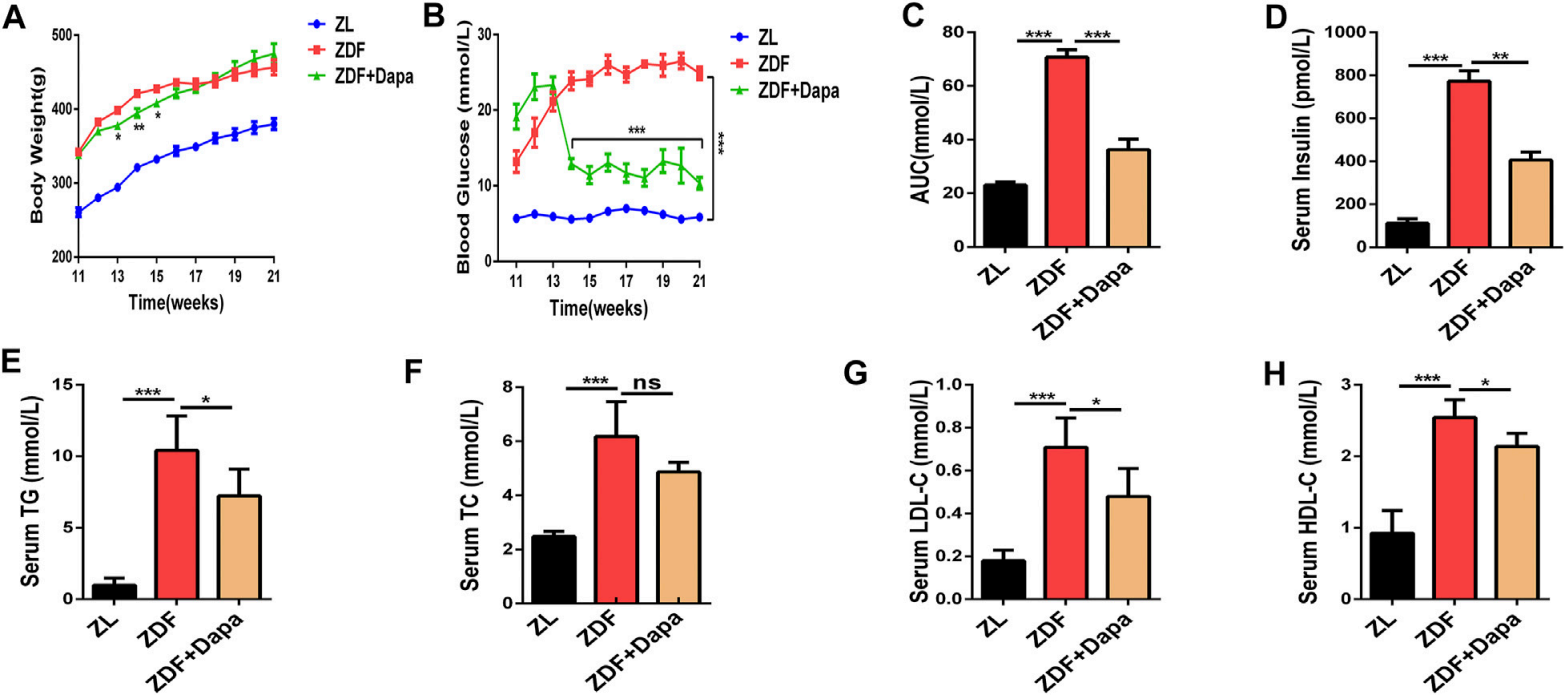
Dapagliflozin improves glucose intolerance and regulates lipid metabolism in ZDF rats. **(A)** Body weight was recorded every week and body weight gain was measured at the end of the 21st week. **(B)** Blood glucose levels were monitored throughout the entire experiment. **(C)** The area under the curve (AUC) of blood glucose was calculated by the trapezoidal method. **(D)** Serum insulin levels were evaluated in groups. **(E–H)** Serum levels of TG, TC, LDL-C, and HDL-C were evaluated in groups. ZL: ZL rats as a normal control, ZDF: ZDF rats fed with a high-fat diet, ZDF + Dapa: dapagliflozin-treated ZDF rats. Data are expressed as the means ± SEM of five animals per group. *p < 0.05, **p < 0.01, and ***p < 0.001 compared with the ZDF rat group.

The ZDF rat is a recognized T2DM animal model characterized with overweight, impaired glucose tolerance and hyperlipidemia due to the leptin receptor mutation (Al-awar et al., 2016)*.* To confirm the influence of dapagliflozin on the metabolic phenotypes of ZDF rats *in vivo*, body weight and blood glucose were weekly assessed in different groups. As shown in Figure 1A, the body weight of ZDF rats was significantly higher than that of ZL rats from 11 to 21 weeks. In the first three weeks after the administration of dapagliflozin, the body weight of ZDF rats began to decrease significantly compared to that of the ZDF rats without treatments. At the end of the experiment, the body weights of the ZDF + Dapa and ZDF groups showed no significant difference. In addition, the blood glucose level in the treatment group was dramatically reduced throughout the entire experiment (Figure 1B). To investigate the effect of dapagliflozin on glucose tolerance, OGTTs were performed on all animals without anesthesia after an overnight fast. As demonstrated in Figure 1C, the AUC revealed that the ZL group was tolerant of glucose, while the rats in ZDF group had an impaired tolerance to glucose. However, the impaired glucose tolerance was significantly improved after dapagliflozin administration. In addition, dapagliflozin treatment also improved whole body insulin sensitivity in this ZDF rat model as reflected by a significant reduction in plasma insulin concentrations (Figure 1D).

To evaluate the effect of dapagliflozin on lipid metabolism, serum lipid profiles including total cholesterol (TC), triglyceride (TG), low-density lipoprotein cholesterol (LDL-C) and high-density lipoprotein cholesterol (HDL-C) levels were tested in groups. Compared with the ZL rats, the ZDF rats exhibited markedly increased serum levels of TC, TG and LDL-C, indicating an impaired lipid metabolism. Compared with the ZDF rats, the ZDF rats with the dapagliflozin treatments showed significant decrease in the abnormally elevated serum TG and LDL-C levels, although the TC levels were not statistically impacted by the dapagliflozin intervention (Figures 1E–H).

### Dapagliflozin Alleviates Hepatic Lipid Accumulation in ZDF Rats and PA-Stimulated LO2 and HepG2 Cells

**FIGURE 2:**
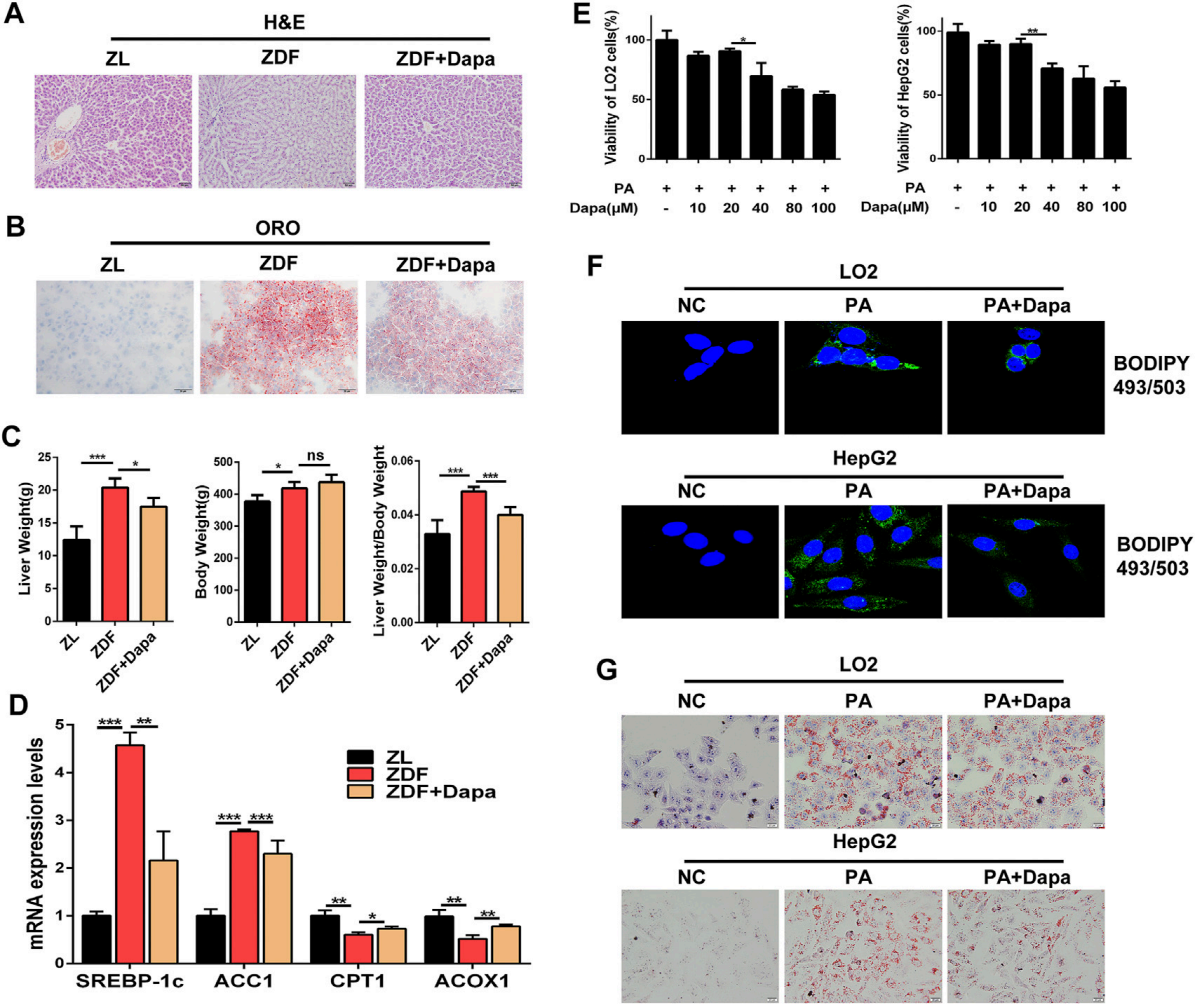
Dapagliflozin alleviates hepatic lipid accumulation in ZDF rats and PA-stimulated LO2 and HepG2 cells. **(A, B)** Liver sections were stained with H&E (Scale bars: 50 μm)and Oil Red O (Scale bars: 20 μm). **(C)** Liver/body weight ratio was calculated by liver weight (g)/body weight (g). **(D)** The mRNA expression levels of hepatic lipogenic genes ACC1, SREBP-1c, and fatty acid oxidation genes CPT1 and ACOX1 were examined by real-time q-PCR **(E)** CCK-8 assays indicate the effect of dapagliflozin (10, 20, 40, 80, and 100 μmol/L) on the viability of HepG2 cells and LO2 cells. **(F)** HepG2 cells and LO2 cells were stained with 1 μg/ml BODIPY 493/503 and fluorescence images were captured by a confocal microscope. **(G)** LO2 and HepG2 cells were stained with Oil Red O. Scale bars: 20 μm. Data are expressed as the means ± SEM from three independent experiments. *p < 0.05, **p < 0.01, and ***p < 0.001.

To determine whether dapagliflozin alleviated hepatic lipid accumulation *in vivo*, the liver/body weight ratio and hepatic lipid accumulation in hepatic histology were evaluated in groups. The histological observation of H&E and Oil Red O staining indicated that dapagliflozin administration apparently alleviated hepatic steatosis through reducing the fatty degeneration of hepatocytes and intracellular lipid droplet formation (Figures 2A,B). As shown in Figure 2C, significant differences in body weight were not observed between the ZDF rats with or without dapagliflozin treatment, but the liver weight and the liver/body weight ratio were dramatically reduced in comparison with those of the ZDF group. Moreover, dapagliflozin treatment downregulated the expression of several hepatic lipogenic genes including ACC1 and SREBP-1c, and upregulated genes related to fatty acid oxidation, such as CPT1 and ACOX1 (Figure 2D).

Next, we examined the effect of dapagliflozin on lipid accumulation in LO2 and HepG2 cells. Prior to testing the effect of dapagliflozin in cells *in vitro*, a CCK-8 assay was performed to evaluate the cytotoxic effects of dapagliflozin on hepatic cells, and the results showed that dapagliflozin had a limited effect on the viability of LO2 cells and HepG2 cells, even at a concentration up to 20 μmol/L (Figure 2E). As shown in Figure 2F, the fluorescence intensity of BODIPY 493/503-stained lipid droplets was decreased after dapagliflozin administration in cells. To further confirm the lipid clearance effects of dapagliflozin in hepatic cells, we stained lipid droplets with Oil Red O (ORO). Exposure to 0.3 mM PA for 24 h dramatically increased the intracellular lipid accumulation in LO2 cells and HepG2 cells, whereas cotreatment with dapagliflozin could obviously attenuate the PA-induced lipid accumulation in both of hepatic cell lines (Figure 2G). Taken together, these findings indicated that dapagliflozin prevented against hepatic lipid deposition in ZDF rats and PA-stimulated hepatic cells.

### Expression of SGLT2 in Rat Liver, LO2 Cells and HepG2 Cells

**FIGURE 3:**
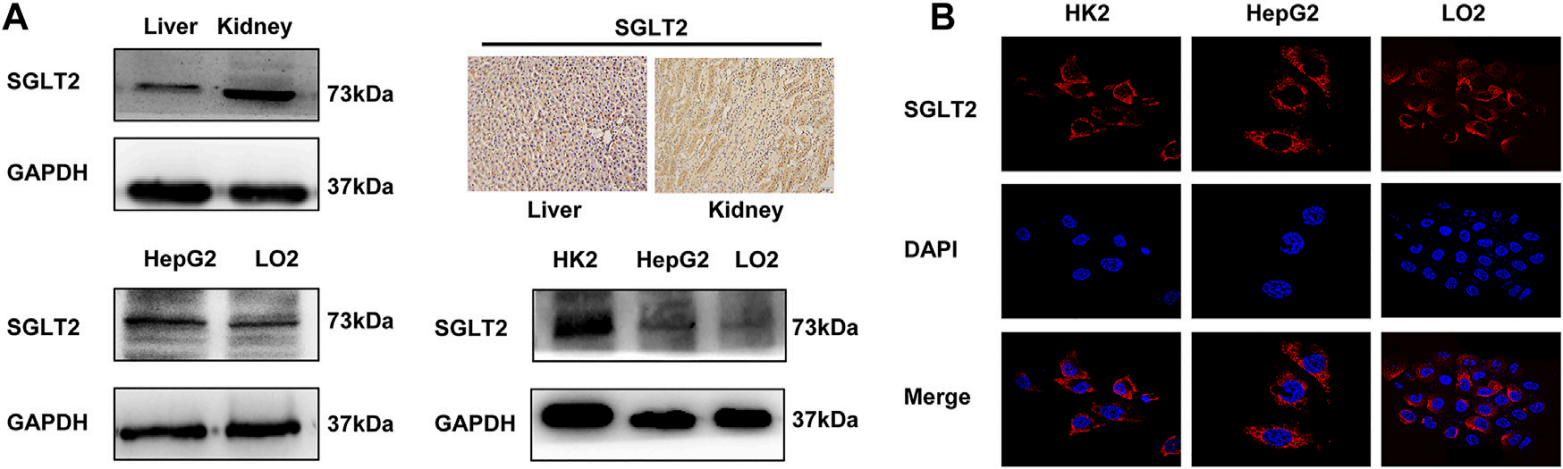
Expression of SGLT2 in rat liver and LO2 and HepG2 cells. **(A)** Western blot analysis and IHC staining demonstrate the protein expression level of SGLT2 in rat liver and kidney tissues. **(B)** Western blot analysis and immunofluorescence staining show the expression of SGLT2 in human hepatic cell lines.

To determine whether dapagliflozin exerted its effects by targeting on SGLT2, we investigated the expression of SGLT2 in LO2, HepG2, HK2 cells, and animal tissues by Western blot, immunofluorescence and immunohistochemistry analyses. As shown in Figure 3A, the Western blot analysis showed a band in the liver with the same molecular weight as that in the kidney, which indicates the expression of SGLT2 in rat liver. Immunohistochemical staining also confirmed the expression of SGLT2 predominantly on the cell membrane in both kidney and liver tissues. In addition, immunofluorescence staining and Western blot analysis indicated the expression of SGLT2 in LO2 cells and HepG2 cells (Figure 3B).

### Dapagliflozin Induces Autophagy and Regulates Fatty Acid Metabolism in ZDF Rats and PA-Stimulated LO2 and HepG2 Cells

**FIGURE 4:**
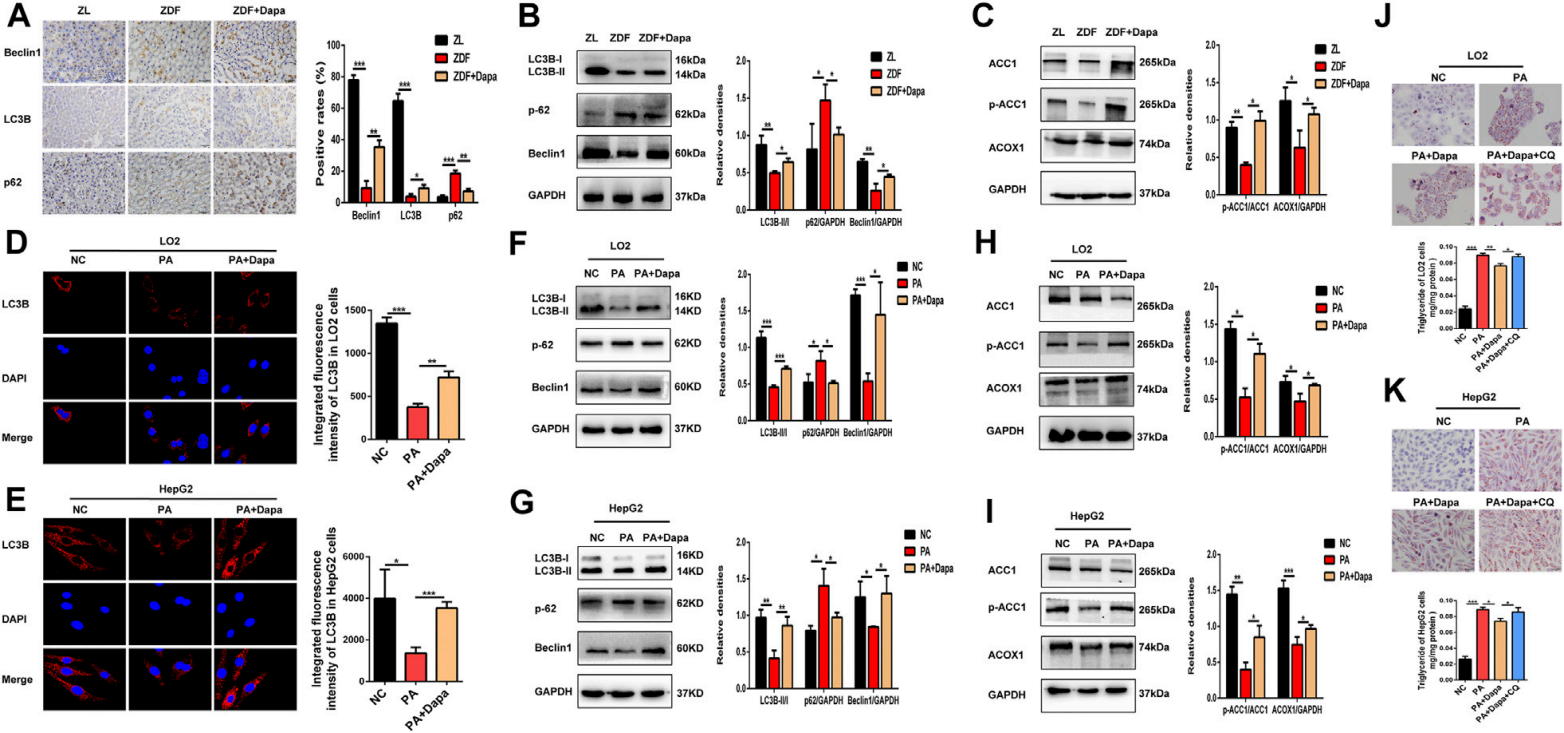
Dapagliflozin induces autophagy and regulates fatty acid metabolism in ZDF rats and PA-stimulated LO2 and HepG2 cells. **(A)** IHC staining detected the expression of LC3B, Beclin-1 and p62 in hepatic tissue. Scale bars: 20 μm. Data are expressed as the means ± SEM of five animals per group. *p < 0.05, **p < 0.01, and ***p < 0.001 compared with the ZDF rat group. **(B)** Western blot analysis evaluated the protein expression levels of LC3B, Beclin-1, and p62 in rats. **(C)** Western blot analysis detected the expression of ACC1, p-ACC1, and ACOX1 in the livers of rats. **(D, E)** LC3B immunofluorescence staining showed the endogenous LC3B level of LO2 and HepG2 cells. **(F, G)** Western blot analysis demonstrated the expression of LC3B, Beclin-1, and p62 in LO2 and HepG2 cells. **(H, I)** Western blot analysis detected the protein expression of ACC1, p-ACC1 and ACOX1 in cells. **(J, K)** LO2 and HepG2 cells were treated with 0.3 mM PA, 20 μM dapagliflozin and 20 µM chloroquine (CQ) for 24 h. The cells were stained with Oil Red O and intracellular TG was quantitatively analyzed. Scale bars: 20 μm. Data are expressed as the means ± SEM from three independent experiments. *p < 0.05, **p < 0.01, and ***p < 0.001.

We examined the autophagic alterations in the rat liver and compared the effects with PA-stimulated LO2 and HepG2 cells. Detection of autophagy and the expression of autophagy-related genes were evaluated by Western blot analysis and immunohistochemistry (IHC) in the liver tissue of each group. Immunohistochemical staining showed that dapagliflozin administration significantly increased the expression of LC3B and Beclin1, but decreased that of p62 in the liver of ZDF rats (Figure 4A). As shown in Figure 4B, dapagliflozin significantly upregulated the levels of LC3B-II and Beclin1 and decreased the levels of p62 compared with the ZDF group. In addition, the administration of dapagliflozin increased ACC1 phosphorylation and significantly upregulated fatty acid oxidation gene ACOX1 (Figure 4C). These data suggested that dapagliflozin induced autophagy and improved cellular lipid metabolism in ZDF rats.

Moreover, the effects of dapagliflozin on the LC3B expression in PA-induced hepatocytes were detected by immunofluorescence staining and Western blot assays. Administration of dapagliflozin dramatically increased the expression of LC3B, as indicated by the enhanced fluorescence intensity (Figures 4D,E). Consistently, the Western blot assays demonstrated that dapagliflozin increased the expression of LC3B-II and Beclin1, and inhibited the expression of p62, which strongly indicated the enhancement of autophagy (Figures 4F,G). Similar outcomes on ACC1 phosphorylation and ACOX1 protein expression were also observed after dapagliflozin administration in PA-stimulated LO2 cells and HepG2 cells (Figures 4H,I). To assess the influence of dapagliflozin on autophagy, we pharmacologically blocked autophagosome-lysosome fusion using chloroquine diphosphate (CQ, 20 μmol/L), a lysosomal inhibitor. As shown in (Figures 4J,K), cotreatment with CQ abolished the effect of dapagliflozin on reducing lipid accumulation, indicating that involvement of autophagy in dapagliflozin alleviated hepatic lipid accumulation.

### Dapagliflozin Induces Autophagy via the AMPK-mTOR Pathway *in vitro* and *in vivo*

**FIGURE 5:**
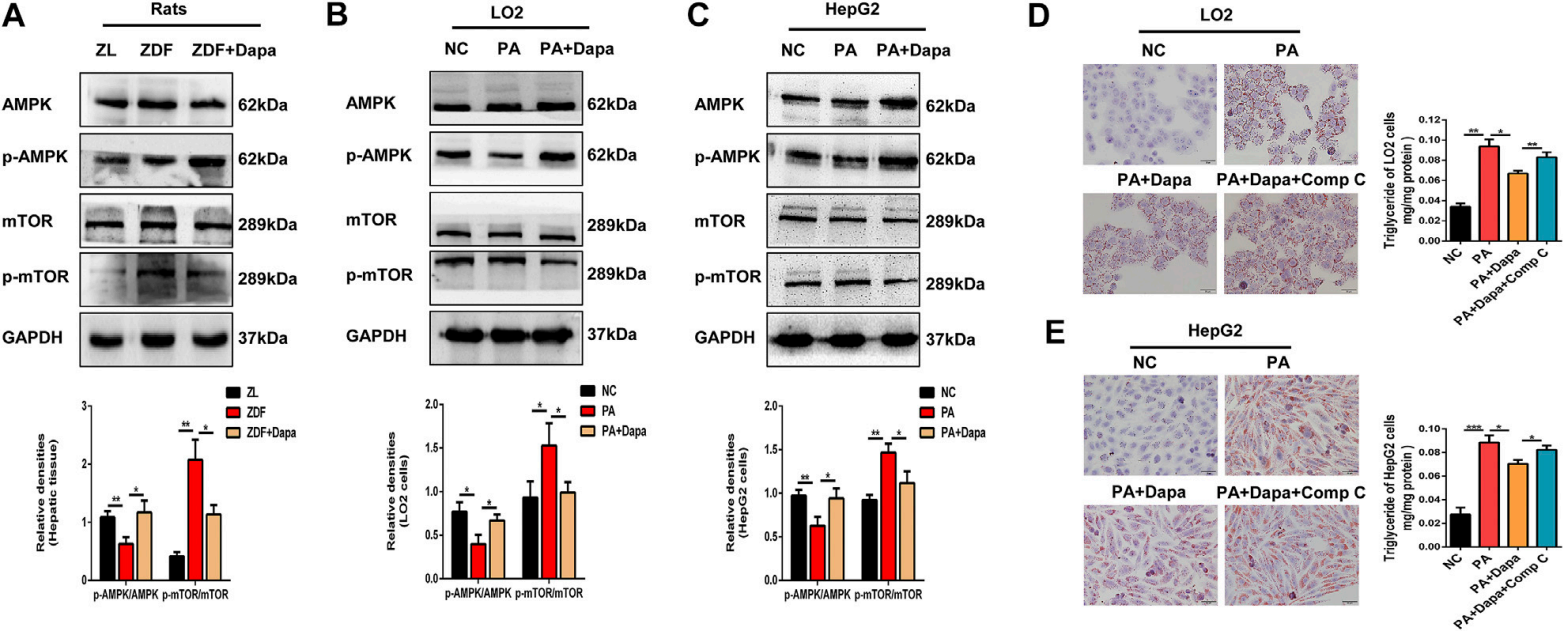
Dapagliflozin induces autophagy via the AMPK-mTOR pathway *in vitro* and *in vivo.* **(A–C)** Western blot analysis evaluated the protein expression levels of p-AMPK, AMPK, p-mTOR, and mTOR in rats and cells. **(D, E)** LO2 and HepG2 cells were treated with 0.3 mM PA, 20 μM dapagliflozin and 10 µM compound C (Comp C) for 24 h. The cells were stained with Oil Red O and intracellular TG was quantitatively analyzed. Scale bars: 20 μm. Data are expressed as the means ± SEM from three independent experiments. *p < 0.05, **p < 0.01, and ***p < 0.001.

To test whether autophagy is activated via the AMPK-mTOR pathway, the protein expression of AMPK and mTOR was examined *in vitro* and *in vivo*. As shown in Figures 5A–C, the p-AMPK/AMPK ratio was elevated in dapagliflozin-treated ZDF rats or hepatocytes, while the p-mTOR/mTOR ratio was significantly reduced. Then, to determine the association of AMPK activation with dapagliflozin-induced autophagy, AMPK inhibitor compound C (Comp C) was used to block AMPK phosphorylation in hepatic cells. Treatment with Comp C reversed the effect of dapagliflozin on cellular lipid deposition, indicating the involvement of the AMPK pathway in dapagliflozin-mediated protection against hepatic steatosis (Figures 5D,E).

## Discussion

NAFLD is a burgeoning health problem worldwide and has become a significant risk factor for both hepatic and cardiometabolic mortality (Al-awar et al., 2016). However, effective drugs approved for the treatment of NAFLD are still lacking. SGLT2 inhibitors are listed as a new class of oral anti-hyperglycaemic medications for the pharmacological management of T2DM (Chaudhury et al., 2017). A single-center retrospective observational study indicated that dapagliflozin and empagliflozin could improve the metabolic and hepatic disorders (Lee et al., 2018). Thus, it is of great significance to explore the underlying specific mechanisms of SGLT2 inhibitors in alleviating the diet-induced metabolic dysfunction and NAFLD. In the present study, we report a beneficial effect of dapagliflozin in ameliorating hepatic steatosis by modulating AMPK-mediated autophagic activation. We observed for the first time that the dapagliflozin alleviated lipid accumulation and lipotoxicity, accompanied by induced autophagy in ZDF rats and PA-induced human hepatic cells, indicating that the antihepatosteatotic effects of dapagliflozin might be independent of its hypoglycaemic activities.

Previous studies have demonstrated that the excessive lipid accumulation can lead to cellular injury and death (Czaja, 2016; Li et al., 2020). In this study, we focused on the effects of dapagliflozin on hepatic steatosis and explored the relevant mechanism using hepatic cells and ZDF rats with obesity, NAFLD and metabolic dysfunction. Both LO2 cells and HepG2 cells were used to study the effects of dapagliflozin with the presence of PA. Our data showed that continuous intervention with dapagliflozin attenuated liver weight, lipotoxicity, dyslipidaemia, impaired glucose tolerance and hepatic lipid deposition in ZDF rats. Meanwhile, BODIPY 493/503 and ORO staining indicated that dapagliflozin prevented against intracellular lipid accumulation in PA-stimulated LO2 cells and HepG2 cells.

SGLT2 is a sodium-glucose transporter that is mainly expressed in the proximal convoluted tubules of the kidney, and its ubiquitous expression has also been detected in other human tissues (Uthman et al., 2018). Some studies have demonstrated that the protein expression of SGLT2 could be detected in the immortalized human primary hepatocytes HuS-E/2 cells, human hepatocellular carcinoma HepG2 cells and mouse hepatic tissue (Hawley et al., 2016; Kaji et al., 2018; Chiang et al., 2020). However, the SGLTs family consists of 12 members, and the expression of these proteins in extrarenal tissues is controversial due to the lack of specific antibodies (Wright et al., 2011). In this study, we demonstrated the expression of SGLT2 in hepatic cell lines and rat liver tissues.

Autophagy is a pathway of lysosome degradation that can ameliorate the state of insulin resistance by regulating cellular lipid metabolism (Amir and Czaja., 2011). Currently, emerging evidence suggests that autophagy may be associated with the pathological and physiological changes of NAFLD (Park and Lee., 2014). Autophagy has been reported to delay the progression of NAFLD and protects against liver injury by decreasing hepatocyte lipid accumulation (Czaja, 2016). However, previous studies have not reported on the role of SGLT2 inhibitors in inducing autophagy and reducing hepatic lipid accumulation by directly targeting hepatocytes. Our data strongly suggest that dapagliflozin administration reduces the intracellular lipid accumulation and activates the autophagy machinery because increased autophagy markers (LC3B and Beclin1 protein levels), and decreased p62 levels were found *in vitro* and *in vivo* ***.*** Most importantly, this improvement effect was abolished by incubation with CQ, a lysosomal function inhibitor, suggesting the involvement of autophagy activation in dapagliflozin-mediated hypolipidaemic effects. Hepatic lipid accumulation is caused in part by increased intracellular *de novo* lipogenesis, in which the ACC1 enzyme catalyzes the first rate-controlling step (Goedeke et al., 2018; Ipsen et al., 2018). Specific knockout of ACC1 reduced *de novo* lipogenesis in hepatocytes and lipid accumulation in the liver of mice (Mao et al., 2006). ACOX1 is the rate-limiting enzyme in peroxisomal fatty acid oxidation (Xiao et al., 2020), and the deficiency of ACOX1 leads to hepatic lipid accumulation, inflammation and fibrosis (Ipsen et al., 2018). Thus, reducing *de novo* lipogenesis or increasing fatty acid oxidation will improve hepatic lipid accumulation. In this study, we demonstrated that dapagliflozin phosphorylated and inactivated ACC1 and increased the expression of ACOX1, which helped to alleviate cellular lipid accumulation *in vitro* and *in vivo.*

AMPK is a crucial metabolic regulator that not only inhibits energy-consuming pathways but also activates the energy-compensating process (Ha et al., 2015). Evidence shows that AMPK plays a critical role in autophagy induction in response to various cellular stresses, such as glucose starvation (Vingtdeux et al., 2010). In the liver, AMPK activation regulates metabolism by increasing catabolic pathways, such as autophagy (Alers et al., 2012) and fatty acid oxidation (Fernandez-Galilea et al., 2014), and decreasing anabolic pathways such as lipid synthesis (Zhou et al., 2001). In addition, mTOR, a downstream target of AMPK, negatively regulates autophagy activity (Kim et al., 2011). Our study demonstrated that dapagliflozin could increase the phosphorylation of AMPK, while suppressing the phosphorylation of mTOR in dapagliflozin-treated ZDF rats and cultured hepatic cells. Meanwhile this phenomenon was reversed by the AMPK specific inhibitor compound C, which strongly suggests that the effects of dapagliflozin on the amelioration in hepatic steatosis are directly mediated through AMPK activation. In addition, these results also illustrate that the AMPK-mTOR pathway plays an important role in the activation of autophagy in steatotic hepatic cells.

**FIGURE 6:**
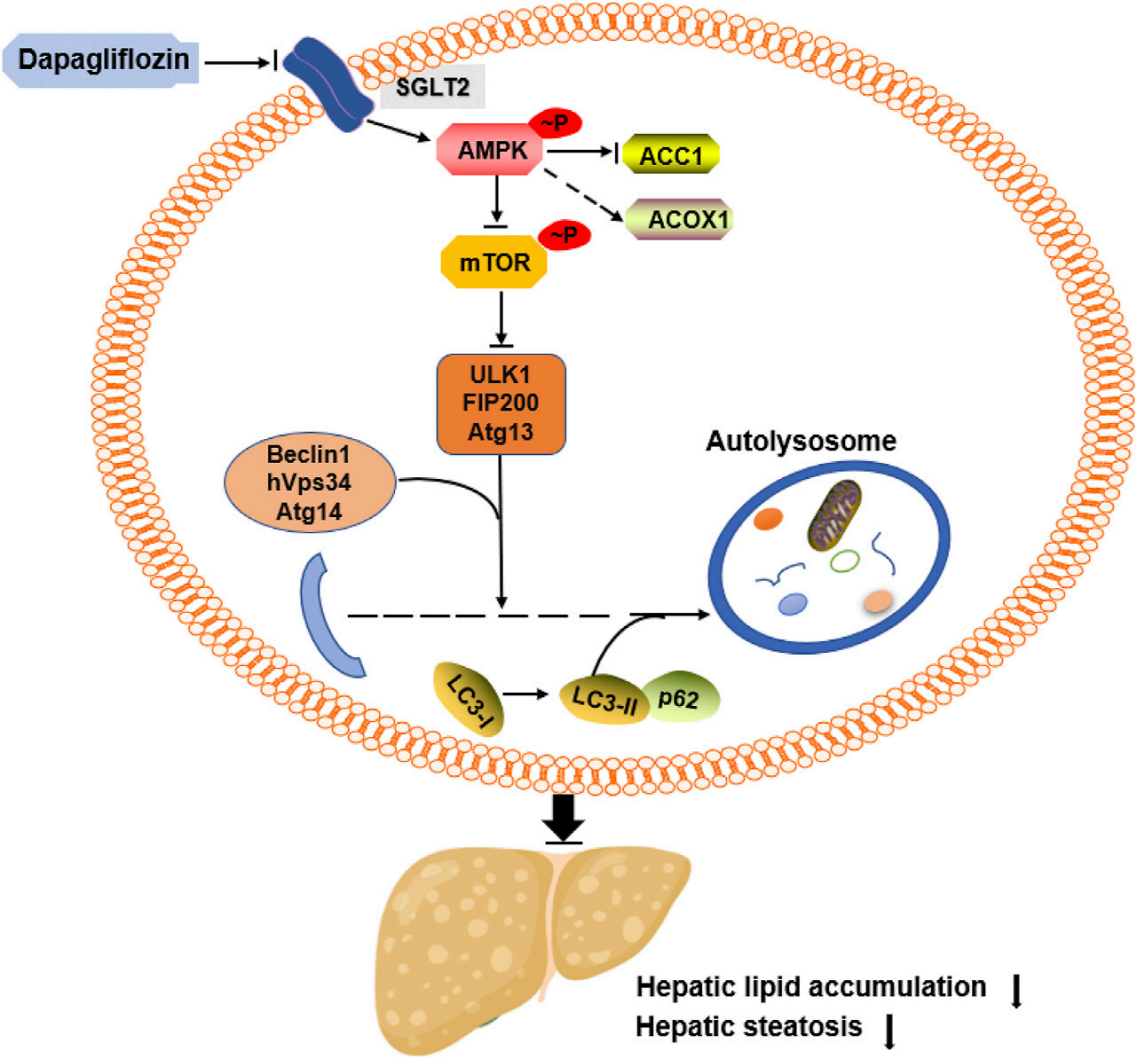
Summary of the underlying mechanism of dapagliflozin on alleviating hepatic steatosis.

In summary, we demonstrated that dapagliflozin ameliorates hepatic steatosis by decreasing the *de novo* lipogenesis enzyme ACC1, increasing the fatty acid oxidation enzyme ACOX1 and inducing autophagy. These beneficial effects seem to be in part mediated through AMPK activation (Figure 6). In addition, our data indicate that dapagliflozin induces autophagy via the AMPK-mTOR pathway. Importantly, our findings suggest an important mechanism for the positive effects of dapagliflozin on alleviating hepatic steatosis and provide evidence for the novel clinical usage of dapagliflozin in NAFLD by targeting intracellular autophagy in hepatic cells.

## Data Availability

All datasets presented in this study are included in the article/Supplementary Material.
