# Supplementary material for: Dapagliflozin Alleviates Hepatic Steatosis by Restoring Autophagy via the AMPK-mTOR Pathway
Source: Front Pharmacol. 2021 May 17;12:589273. doi: 10.3389/fphar.2021.589273 (PMC8176308; doi:10.3389/fphar.2021.589273)
Supplement: Supplementary file 1 [file Table1.DOCX]

**Supplementary Table 1** Primers used for quantitative real-time PCR.

| Gene | Forward primer(5’ to 3’) | Reverse primer(5’ to 3’) |
| --- | --- | --- |
| ACC1 | TGGATTTTTTGATTATGGCTCTTTC | CCTGGCTCTGCCAACTACCA |
| SREBP-1c | CAGGTCCTTGAGCTCCACAAT C | GCCCACAATGCCATTGAGA |
| CPT1  ACOX1 | CCTTGGCTACTTGGTACGAAT TCT  CTTCAGGTAGCCATTATCCATCTCT | GCGGATGCAGTGGGACAT  GCCTTTGTTGTCCCTATCCGT |
